# Experience With the Use of the MicroDTTect Device for the Diagnosis of Low-Grade Chronic Prosthetic Joint Infections in a Routine Setting

**DOI:** 10.3389/fmed.2021.565555

**Published:** 2021-03-16

**Authors:** Camille Kolenda, Jérôme Josse, Cécile Batailler, Allison Faure, Alice Monteix, Sébastien Lustig, Tristan Ferry, Frédéric Laurent, Céline Dupieux

**Affiliations:** ^1^CIRI, Centre International de Recherche en Infectiologie, Inserm U1111, Université Claude Bernard Lyon 1, CNRS UMR5308, ENS de Lyon, Lyon, France; ^2^Centre de Référence des Infections Ostéo-Articulaires Complexes de Lyon, Lyon, France; ^3^Laboratoire de Bactériologie, Institut des Agents Infectieux, Hôpital de la Croix-Rousse, Hospices Civils de Lyon, Lyon, France; ^4^Service de Chirurgie Orthopédique, Hôpital de la Croix-Rousse, Hospices Civils de Lyon, Lyon, France; ^5^Service de Maladies Infectieuses et Tropicales, Hôpital de la Croix-Rousse, Hospices Civils de Lyon, Lyon, France

**Keywords:** dithiothreitol, prosthetic joint infection, bone and joint infection, microbiological diagnosis, biofilm, implant failure

## Abstract

**Background:** In prosthetic joint infections (PJIs), identification of the causative microorganisms is critical to successfully adapt and optimize treatment. However, microbiological diagnosis of PJIs remains a challenge notably because bacteria are embedded in biofilm adhered to the prosthetic material. Recently, dithiothreitol (DTT) treatment of prosthesis has been proposed as a new strategy to release bacteria from biofilm and to improve the yield of microbiological diagnosis. In this study, we evaluated the interest of a commercial device using DTT, the MicroDTTect system (Heraeus, Hanau, Germany), for the diagnosis of low-grade chronic PJIs, compared to the conventional culture of periprosthetic tissue (PPT) samples.

**Methods:** Twenty patients undergoing a surgery procedure for removal of prosthetic material because of a suspicion of low-grade PJI without pre-operative microbiological documentation were included (NCT04371068). Bacteriological results using the fluid obtained after prosthesis treatment with the MicroDTTect system were compared to results obtained with conventional culture of PPT samples.

**Results:** All the bacteria considered as responsible for PJIs recovered from culture of PPT samples were also detected using the MicroDTTect device. For one patient, an additional bacterial isolate (*Staphylococcus haemolyticus*) suspected to be involved in a polymicrobial PJI was identified using DTT treatment. Time to positivity of the cultures was also reduced using the MicroDTTect system, notably in case of *Cutibacterium acnes* infection. However, probable bacterial contaminants were found (MicroDTTect system, *n* = 5; PPT samples, *n* = 1).

**Conclusion:** This study showed that DTT treatment of the prosthetic component using the MicroDTTect device could improve the microbiological diagnosis of low-grade PJIs.

## Introduction

Prosthetic joint infections (PJIs) are one of the major causes of implant failure after joint arthroplasty with an average incidence of 1.1% for primary total hip replacement and 0.6% for primary total knee replacement (1). These infections are associated with high morbidity rates and health-care costs (2). The diagnosis of PJIs can be very challenging, notably in case of infection due to low-virulence organisms, as clinical features and microbiological diagnostic results may be conflicting (3). A definition of periprosthetic infection has been proposed by the MusculoSkeletal Infection Society (MSIS), including major and minor criteria (4). However, in some forms of low-grade PJI, several of these criteria may not be met despite the presence of a true infection, such as the identification of the microorganism involved after culture of periprosthetic tissue (PPT) samples, whereas it is pivotal for antimicrobial susceptibility tests and subsequently for treatment adaptation. Among others, biofilm formation, which corresponds to a complex microbial community adherent to various surfaces and protected by self-produced matrix, is a major limiting factor for culture of bacterial pathogens in the context of PJIs (5). Traditional sampling techniques may fail to detach viable biofilm-embedded bacteria from prosthetic surfaces and periprosthetic tissues, thus leading to false negative results in culture and to a possible diagnostic conclusion of aseptic failure, especially when clinical signs are confounding, as in low-grade infections or in case of recent antibiotic treatment.

To overcome these difficulties, different methods have been developed to improve the microbiological diagnosis yield in PJIs by disrupting the bacterial biofilm before culture. For instance, superiority of culture of sonication fluids obtained from explanted prosthesis over conventional culture of PPT samples has been reported in several studies (6–9). However, sonication is limited by the need of specific instrumentation, which is neither available nor affordable in all laboratories, the difficulties in managing large implants, and the risk of sample contamination, caused by improperly sealed sample containers and/or bacteria proliferation in sonication water. Recently, an alternative method for biofilm detachment using a chemical agent, namely DL-dithiothreitol (DTT), has been proposed (10). DTT is a sulfhydryl compound that reduces disulfide bonds and destroys intra- and inter-molecular bonds between cysteine residues in proteins. It can alter the extracellular matrix of biofilm and release bacteria from it, without affecting bacterial viability allowing further bacterial growth before identification and antibiotic susceptibility testing with traditional methods (11). Drago et al. notably showed that DTT treatment could be a reliable alternative to sonication for the microbiological diagnosis of PJIs because it is easier to use, as the procedure does not require any specific laboratory instruments and it was associated with a better sensitivity and the same specificity compared to sonication (12).

A commercial device containing a DTT solution has been developed, namely MicroDTTect (4i for infection, Monza, Italy), to simplify the process, reduce the multiple transfers and steps with technical manipulations, and thus to limit risks of contamination. To evaluate the added value of this approach, we focused our study to patients presenting with a suspicion of low-grade chronic PJI, clinical context in which the MicroDTTect system could be particularly of interest compared to conventional culture of PPT samples.

## Materials and Methods

### Patient Recruitment

Between February 2018 and August 2019, a total of 20 patients undergoing a surgery procedure for prosthetic material removal in the surgery department of Croix Rousse hospital (Lyon University Hospital, CRIOAc Lyon, France) because of painful prosthesis, prosthesis loosening happening <10 years after the implantation, or suspicion of chronic PJI with negative joint puncture or with no joint puncture performed in the previous 3 months, were enrolled in this study. Exclusion criteria included mechanical explanation for the pain or loosening, and clinical evidence of infection (fistula, abscess, discharge, or local inflammation). The study group included 12 men and 8 women, undergoing surgery for total knee (*n* = 15) or hip (*n* = 5) prosthesis replacement (one- or two-stage).

### Samples Collection

**Figure 1:**
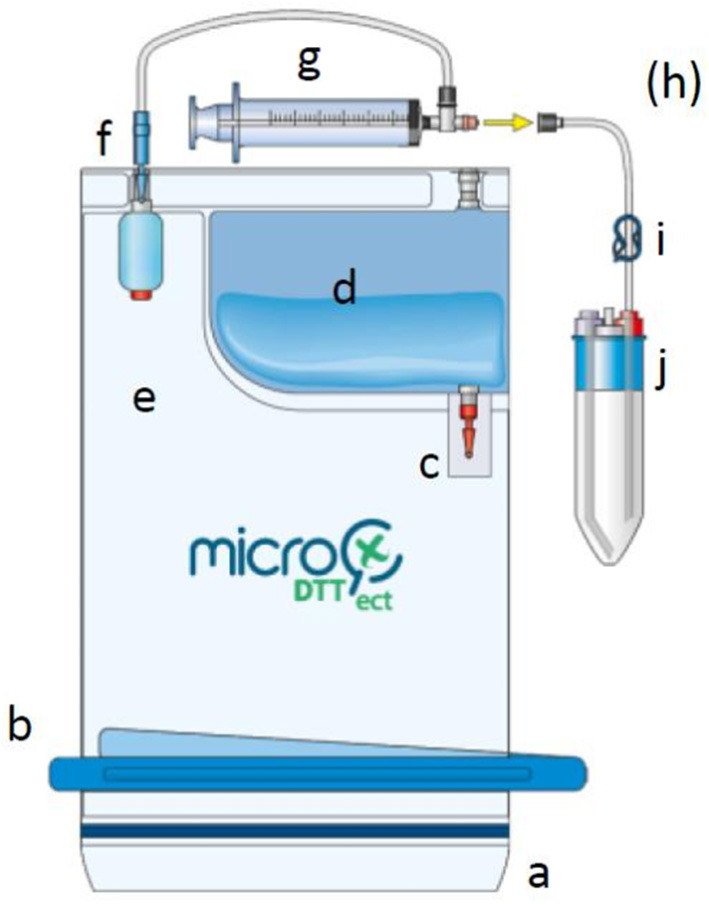
Drawing showing the different components of the MicroDTTect device. The MicroDTT system is composed of: (a) closing system with minigrip, (b) clamp for the second closure, (c) unbreakable red valve, (d) dithiothreitol solution at 0.1% (w/v), (e) PVC two-compartment bag, (f) unbreakable blue valve, (g) syringe, (h) injection point with needle (optional, for direct inoculation into blood culture bottles), (i) blue clip, and (j) liquid collection test tube(s). Prosthetic implant was aseptically collected in the operating room and immediately placed in the MicroDTTect device (compartment e). After mechanical agitation, the DTT suspension was transferred into several sterile tubes (j). This figure is published with the permission of Heraeus.

Prosthetic implants were aseptically collected in the operating room and immediately placed in the MicroDTTect collection system (Figure 1). In parallel, PPT samples (between 5 and 7 per patient) were also collected according to the usual protocols of the hospital and put into plastic sterile containers Ultra-Turrax® (Labelians, Nemours, France) containing metallic beads, immediately sealed and transported to the laboratory for standard microbiological analysis.

### Microbiological Procedures

The MicroDTTect procedure was performed according to the manufacturer's recommendations. The MicroDTTect device containing the prosthesis was placed on a mechanical shaker for 15 min to increase contact between DTT and prosthesis, in order to detach bacteria and biofilm from the material surface. The obtained DTT suspension was transferred into dedicated test tubes. Three BACT/ALERT® aerobic FA Plus, anaerobic FN Plus, pediatric PF plus blood culture bottles (bioMérieux) were inoculated with 5 mL of this suspension. The tubes were then centrifuged at 3,200 rpm for 10 min and then, all the supernatant except 1 mL was discarded. After resuspension of the pellet in the remaining DTT solution, 100 μL were plated onto: one sheep blood agar plate (bioMérieux, Marcy l'Etoile, France) incubated during 2 days in aerobic atmosphere, two chocolate blood agar plates (bioMérieux) incubated for 2 and 5 days under 5% CO_2_, and two Schaedler agar plates (bioMérieux) incubated for 5 and 14 days in anaerobic conditions. In addition, one Schaedler broth was also inoculated with 100 μL of the resuspended pellet. Bacterial growth was followed automatically by a BACT/ALERT® VIRTUO® system (bioMérieux) for the bottles and every day visually for the broth during 14 days. If positive, broth or bottles were subcultured onto one sheep blood agar, one chocolate agar, and one Schaedler agar plates incubated for 2 days in CO_2_ and anaerobic condition, respectively.

In parallel, PPT samples were homogenized using a specific grinder Ultra-Turrax® Tube Drive Ika® (Labelians) during 1 min followed by plating of 20 μL on the same solid agar media as described above and inoculation of 100 μL into a Schaedler liquid broth.

When cultures were positive, each morphology of colony was identified by matrix-assisted laser desorption ionization time-of-flight mass spectrometry using the VITEK® MS device (bioMérieux). Results obtained with both approaches (MicroDTTect device and PPT samples) were compared in terms of number and types of bacterial species, time to positivity, and semi-quantitative analysis of the bacterial inoculum in the samples (number of colonies on plates).

### Ethics

For this observational study, which did not require the approval of an ethics committee, written information was given to all patients. The study was registered as a clinical trial (no. NCT04371068) and was approved by the National Data Protection Commission (no. 18–265), as required by national ethics rules.

## Results

Bacteriological growth was observed for 11 out of 20 (55%) prosthetic samples treated using the MicroDTTect system, while PPT samples were positive in culture for only 8 out of 20 patients (40%) (Table 1). Isolated bacteria were mostly Gram-positive cocci: coagulase-negative staphylococci (n=10 strains/8 patients using MicroDTTect; *n* = 4 strains/4 patients in PPT samples) and *Enterococcus faecalis* (1 strain in both DTT and PPT samples). *Cutibacterium acnes* isolates were also recovered for two patients with both techniques, and one additional isolate was found in one PPT sample.

**Table 1:** Comparison of microbiological results obtained by culture of the prosthetic material using the MicroDTTect device vs. classical culture of periprosthetic tissue samples.

**Patient**	**Type of surgery**	**Routine lab results**				**MicroDTTect results**				**Final diagnosis of infection (Yes/No)**
		**Bacterial species**	**No. of pos samples**	**Time to positivity**	**No. of col/plate**	**Bacterial species**	**No. of pos media**	**Time to positivity**	**No. of col/plate**	
1	2-stage TKP replacement	*S. caprae*	5/5	24 h	EBO-50 CFU	*S. caprae*	9/9	24 h	50 CFU	Y
2	2-stage TKP replacement	–	0/5	–	–	–	–	–	–	N
3	1-stage TKP replacement	–	0/6	–	–	–	–	–	–	N
4	1-stage THP replacement	–	0/5	–	–	–	–	–	–	N
5	1-stage TKP replacement	–	0/5	–	–	–	–	–	–	N
6	2-stage THP replacement	*S. epidermidis*	2/5	48 h	1 CFU	*S. epidermidis*	9/9	24 h	100 CFU	Y
7	2-stage TKP replacement	*S. epidermidis*	5/5	48 h	2–10 CFU	*S. epidermidis*	9/9	24 h	25–100 CFU	Y
8	2-stage THP replacement	*E. faecalis*	4/5	48 h	EBO-4 CFU	*E. faecalis*	9/9	24 h	100–200 CFU	Y
9	2-stage THP replacement	*C. acnes*	2/5	14 days	10 CFU	*C. acnes*	4/9	5 days	100 CFU	Y
10	2-stage TKP replacement	*S. epidermidis*	4/5	48 h	EBO-10 CFU	*S. epidermidis* *S. haemolyticus*	9/9 9/9	24 h 24 h	200–300 CFU 200–300 CFU	Y
11	1-stage TKP replacement	–	0/5	–	–	*S. epidermidis* *S. pettenkoferi*	1/9 1/9	5 days 5 days	10 CFU 10 CFU	N
12	1-stage THP replacement	*C. acnes*	4/5	14 days	1–10 CFU	*S. epidermidis* *C. acnes*	1/9 7/9	48 h 3 days	5 CFU 200–500 CFU	Y
13	1-stage TKP replacement	–	0/7	–	–	*S. epidermidis*	1/9	7 days	50 CFU	N
14	1-stage TKP replacement	–	0/5	–	–	–	–	–	–	N
15	1-stage TKP replacement	–	0/5	–	–	–	–	–	–	N
16	1-stage TKP replacement	*C. acnes*	1/5	14 days	1 CFU	–	–	–	–	N
17	1-stage TKP replacement	–	0/5	–	–	–	–	–	–	N
18	1-stage TKP replacement	–	0/5	–	–	–	–	–	–	N
19	1-stage TKP replacement	–	0/5	–	–	*S. haemolyticus*	1/9	7 days	5 CFU	N
20	1-stage TKP replacement	–	0/5	–	–	*M. luteus*	1/9	30 h	EBO	N

*Pos, positive; col, colonies; CFU, colony-forming unit; EBO, enrichment broth only*.

Agreement between the results obtained with MicroDTTect prosthesis processing vs. conventional culture of PPT samples was observed for 13 out of 20 patients (65%) (same bacteria identified, *n* = 5; negative culture, *n* = 8). For seven out of eight patients with culture-positive PPT sample(s), bacteria recovered from PPT samples were also detected using the MicroDTTect procedure. However, for two patients, if one species was recovered using both approaches (*Staphylococcus epidermidis* and *C. acnes*, respectively), an additional species was collected only using the MicroDTTect procedure: for patient n°10, numerous colonies of *S. haemolyticus* grew on all culture media using the MicroDTTect procedure and was considered as a likely pathogen involved in a polymicrobial infection, undetected with the conventional culture of PPT samples; for patient n°12, *S. epidermidis* was recovered on only one out of the nine inoculated media (five colonies). For patient n°16, a single colony of *C. acnes* was found for only one out of five PPT samples, while the MicroDTTect culture was negative and was considered as a contaminant by clinicians. For four patients (n°11, 13, 19, and 20), likely skin flora contaminants (coagulase-negative staphylococci, *Micrococcus luteus*) were isolated after a culture of MicroDTTect suspension on one out of the nine media, while the culture of PPT samples remained negative. For these, patient records were discussed during a multidisciplinary meeting, which concluded that there was no evidence of PJI based on bacteriological and histopathological analyses of PPT samples.

When considering only likely true culture positive samples, time to positivity using the MicroDTTect system was at least equivalent to PPT culture and, in most cases (six out of seven), decreased by 24 h and up to 9 and 11 days for patients with a true *C. acnes* infection (n°9 and 12, respectively). Of note, the bacterial inoculum recovered after the culture of the prosthetic material was also higher than the one obtained with conventional culture of PPT samples (Table 1).

## Discussion

Diagnosis of PJIs remains a major challenge for microbiology laboratories. Despite the continuous development of innovative microbiological techniques, none of them is considered as a definitive gold standard, and the diagnosis of bone and joint infections is based on a combination of clinical, biological, and/or microbiological arguments. Biofilm formation during chronic PJI is considered as one of the reason of the insufficient sensitivity of classical culture approach using PPT samples, especially when patients received antimicrobial chemotherapy before sampling (7). In order to improve the accuracy of microbiological diagnostic methods, specific approaches allowing detachment of bacteria from biofilm formed and stuck on prosthetic implants have been developed in the last decade including mechanical (low-frequency ultrasounds) and chemical treatment (DTT). In this study, we evaluated a commercial standardized system using DTT, the MicroDTTect device for the diagnosis of low-grade PJIs, in comparison with conventional culture of PPT samples. The data obtained showed that this device could be a valuable tool for the diagnosis of such infections and to improve the yield of microbiological diagnosis. All bacteria responsible of true PJI (same bacteria isolated at least in two different PPT samples from the same patient) were detected using culture of the prosthesis placed in the MicroDTTect device. Moreover, for one patient, the use of MicroDTTect allowed us to detect a likely polymicrobial infection due to *S. epidermidis* and *S. haemolyticus*, while only *S. epidermidis* was isolated with conventional culture of PPT samples. The results also showed that this device could be of interest to reduce significantly the time to positivity of culture and thus accelerate the microbiological diagnosis and optimize the management of patients, notably in case of infection by low-virulent bacteria, such as *C. acnes*.

A few other studies highlighted that treatment with DTT could be of interest for the diagnosis of PJIs (13–15). Calori et al. reported a higher sensitivity using the same MicroDTTect device for collection of both prosthesis and PPT samples compared to conventional cultures (15). However, the comparative method used consisted in the collection of flocked swabs during the surgery, specimens that are not recommended by the various international guidelines for the diagnosis of PJIs due to lack of sensitivity (16, 17). This could have introduced a major bias in the results. Sambri et al. compared DTT and sonication treatment of prosthetic material in a large cohort of patients undergoing prosthesis revision and showed that both technics were more sensitive than PPT sample cultures (13). Moreover, DTT treatment was superior to sonication among patients in whom infection was not suspected preoperatively, whereas the authors did not observe any difference between DTT and sonication in patients with suspicion of infection before surgery. Finally, in the study of De Vecchi et al., treatment of PPT samples with DTT allowed an increase in sensitivity (14). However, homogenization of samples not treated with DTT was simply performed in sterile saline, without grinding them using beads, which is well-known to improve the microbiological diagnosis of PJI (18).

Contrary to the studies previously mentioned, we chose to evaluate the interest of the MicroDTTect device in a specific subgroup of patients with PJI, namely low-grade PJI, and so excluding patients for whom classical approach for microbiological diagnosis, that is, culture of PPTs, is likely enough sensitive. So, we decided not to include patients if the diagnosis of PJI was certain or strongly suspected, for example, if they presented a fistula communicating with the prosthesis or if a microbiological evidence of infection was available before the surgery. Indeed, we believe that this kind of diagnostic technologies, which trigger additional costs to the classical technics used for PJI diagnosis, should be dedicated to patients for whom the diagnosis is the most difficult and for whom supplementary technics could improve the sensitivity of conventional PPT samples culture. This point of view is also supported by the data of Sambri et al. showing that DTT treatment of prosthetic material was especially relevant when PJI was not suspected preoperatively, that is to say for patients with low-grade infections, presenting no clinical and biological signs of infection. These restrictive criteria account for the limited number of patients included, which is a limitation of the present study. The strict selection of patients also probably explains the absence in our study of some bacterial species frequently involved in PJIs, such as *Staphylococcus aureus* and Gram-negative bacilli. We mainly recovered coagulase-negative staphylococci and *C. acnes* isolates, frequently involved in delayed chronic and low-grade PJIs (19).

The major advantages of the MicroDTTect system are that it is quick and easy to use. It is also a closed system providing a completely sterile transportation of the prosthesis and theoretically reducing the risk of sample contamination as minimal manipulations are required. However, cultures of the MicroDTTect samples of five patients in the present study showed growth of one or two skin commensal coagulase-negative staphylococci, but recovered on only one out of nine seeded media, while culture of PPT samples remained negative. These isolates were considered as likely contaminants. However, it is impossible to know if these contaminations have been “acquired” during manipulation of the prosthesis or during plating. Other studies reported good specificity rates, comparable to those of conventional culture, but some of the authors considered that samples were positive only if at least five colonies grew on agar plates after 24 h, whereas this threshold has not been validated by other studies (12, 13, 15). Finally, Romano et al. also suggested that the use of the MicroDTTect device may allow a substantial economic balance or advantage (20). Although the use of MicroDDTect induces an increase of the direct costs compared to culture of PPT samples, the authors showed that this technology was cost-effective notably thanks to the reduction of the time required for sample treatment and the improvement of diagnostic accuracy compared to tissue cultures combined or not with sonication. The present study was a non-interventional study (results not provided to the clinicians) and did not allow us to evaluate the medico-economic impact of this device. Nevertheless, the impact of such diagnostic tools on the cost of PJIs management deserves to be evaluated more deeply in further studies because the results may be highly variable from one country or hospital to another. These studies should focus on low-grade infections, infections for which the diagnosis may be difficult and for which the MicroDTTect device could represent a real gain in diagnostic sensitivity.

In conclusion, in this study, we showed that treatment of the prosthetic component with DTT using the MicroDTTect device improves the microbiological diagnosis of low-grade PJIs by allowing the identification of additional bacteria and reducing the time required to detect them. For optimal interpretation of results, only patients with several positive media should be considered as infected. The added economic value of this diagnostic device has now to be evaluated in real-life conditions.

## Data Availability Statement

All data supporting the findings of the study are included in the article/supplementary materials, further inquiries can be directed to the corresponding author/s.

## Ethics Statement

Ethical review and approval was not required for the study on human participants in accordance with the local legislation and institutional requirements. Written informed consent for participation was not required for this study in accordance with the national legislation and the institutional requirements.

## Author Contributions

JJ, CD, TF, and FL supervised the project. AF and AM carried out the bacteriological analyses. CB, SL, and TF included the patients. CK analyzed the results and wrote the manuscript with support from CD and FL. All authors discussed the results and contributed to the final manuscript.

## Lyon Bone and Joint Study Group (List of Collaborators)

**Coordinator**: *Tristan Ferry*; **Infectious Diseases Specialists**—*TF, Florent Valour, Thomas Perpoint, Patrick Miailhes, Florence Ader, Sandrine Roux, Agathe Becker, Claire Triffault-Fillit, Anne Conrad, Cécile Pouderoux, Nicolas Benech, Pierre Chauvelot, Marielle Perry, Fatiha Daoud, Johanna Lippman, Evelyne Braun, Christian Chidiac*; **Surgeons**—*Elvire Servien, Cécile Batailler, Stanislas Gunst, Axel Schmidt, Matthieu Malatray, Eliott Sappey-Marinier, Michel-Henry Fessy, Anthony Viste, Jean-Luc Besse, Philippe Chaudier, Lucie Louboutin, Quentin Ode, Adrien Van Haecke, Marcelle Mercier, Vincent Belgaid, Arnaud Walch, Sébastien Martres, Franck Trouillet, Cédric Barrey, Ali Mojallal, Sophie Brosset, Camille Hanriat, Hélène Person, Nicolas Sigaux, Philippe Céruse, Carine Fuchsmann*; **Anesthesiologists**—*Frédéric Aubrun, Mikhail Dziadzko*, and *Caroline Macabéo*; **Microbiologists**—*Frédéric Laurent, Laetitia Beraud, Tiphaine Roussel-Gaillard, Céline Dupieux, Camille Kolenda, Jérôme Josse*; **Pathologist**—*Alexis Trecourt*; **Imaging**—*Fabien Craighero, Loic Boussel, Jean-Baptiste Pialat, Isabelle Morelec*; **PK/PD specialists**—*Michel Tod, Marie-Claude Gagnieu, Sylvain Goutelle*; **Clinical research assistant and database manager**—*Eugénie Mabrut*.

## Conflict of Interest

The authors declare that the research was conducted in the absence of any commercial or financial relationships that could be construed as a potential conflict of interest.
